# Effective Interventions on Improving Elderly's Independence in Activity of Daily Living: A Systematic Review and Logic Model

**DOI:** 10.3389/fpubh.2020.516151

**Published:** 2021-02-15

**Authors:** Mohadeseh Motamed-Jahromi, Mohammad Hossein Kaveh

**Affiliations:** ^1^Department of Health Promotion, School of Health, Shiraz University of Medical Sciences, Shiraz, Iran; ^2^Research Center for Health Sciences, Institute of Health, Department of Health Promotion, School of Health, Shiraz University of Medical Sciences, Shiraz, Iran

**Keywords:** independent living, systematic review, elderly, aged, activity of daily living

## Abstract

This systematic review aimed to investigate the types and characteristics of effective interventions when improving the independence of the elderly during activities of daily living. After developing a search strategy, the various databases, including PubMed, Scopus, Cochrane Library, Science Direct, Proquest, and Embase, were searched up to October 16, 2019. The Review Manager 5.1 software was used to determine the risk of bias. The randomized clinical trials were reviewed to find if their interventions' main goal was to improve the elderly's independence in activities of daily living. Data were extracted independently by two authors. Eight randomized controlled trials were included in the final analysis. Three types of interventions were identified and categorized as cognitive training, physical exercises, and multicomponent interventions. All reviewed studies provided evidence of the effectiveness of interventions in improving older people's ability to perform the activities of daily living. However, the lack of uniform measurement indicators to identify and compare the most effective interventions forced us to develop a conceptual framework for designing future interventional research. This conceptual framework included designing tailored interventions, creating an age-friendly environment as well as financial, psychological, and social support. The proposed conceptual framework can also help develop future systematic reviews focusing on a particular intervention type.

## Introduction

Independence among the elderly when performing individual and social tasks is a very challenging issue in all societies. Independence is considered self-determination, emancipation from coercion, and freedom of thought, selection, and performance (1, 2). Independence among the elderly is defined as the degree of individual autonomy in meeting their daily needs (eating, dressing, showering, etc.) and their right to choose (3). During the aging process, implicit physical, physiological, psychological, and social changes generate disability in activities of daily living and reduce independence (4, 5). Activities of daily living include Basic Activities of Daily Living (BADLs) and Instrumental Activities of Daily Living (IADLs) (6). BADLs include self-care skills such as bathing, dressing, eating, and IADLs, including more complex activities such as using the public transport system, financial management, and buying (7). Decreased independence in the elderly has unpleasant consequences and can cause dependence on others in activities of daily living (ADLs), reduce quality of life, and damage health (8). So it is crucial to maintain the elderly's ability and independence in ADLs (9).

A review of health promotion initiatives showed that various interventions with varying degrees of effectiveness were examined to enhance the elderly's activities of daily living in different studies. Some studies have focused on physical activity, especially mobility exercise and functional training, to improve muscle strength, balance, and ADL (10, 11). Some other studies have measured the effect of cognitive interventions on improving the elderly's performance in ADL (12, 13). A cross-sectional study has also documented that the living environment changes can facilitate the activity and presence of the elderly in the community and thus help increase their independence (14). The question is what kind of interventions can help promote the elderly's independence in activities of daily living and the characteristics of the successful interventions in ADL independence?

In the last decade, several systematic reviews have focused on the elderly's physical functioning and quality of life. Still, their primary purpose was not to investigate effective interventions in terms of the elderly's ADL independence and measure it (10, 11, 15–17). The systematic review of Beswick et al. (18) was the only study to examine the impact of community-based interventions on maintaining the elderly's physical activity and independence. This study has reported a combination of interventions, including education and counseling, fall prevention, community-based care, and interventions aiming to change the elderly's physical and social environment. Still, it did not include cognitive and physical interventions (18). Although various interventions have been reported in the literature to improve the elderly's ability to perform ADLs, it seems most healthcare providers have focused on physical interventions in practice. Therefore, a systematic review of articles can provide them with a list of effective interventions. Consequently, this review was conducted to identify the types and the characteristics of interventions effective in promoting the elderly's independence in practicing their activities of daily living.

## Methods

This systematic review, which was designed in 2019, is based on the Preferred Reporting Items for Systematic Reviews and Meta-Analysis checklist (PRISMA) (19).

We adopted a four-step search strategy to identify relevant articles. First, the PICo strategy based on the Joanna Briggs Institute (20) was used for a research question defined as what kind of interventions were used to increase the elderly's independence in daily living activities in randomized controlled trials? After that, the PICo framework contributed to the definition of the inclusion criteria—Population: elderly; Intervention: cognitive, physical, and environmental interventions; Context: dwelling in the community; and Outcome: independence in ADL. Second, the search was conducted without a time limit up to October 16, 2019 in PubMed, Cochrane trial, Scopus, Science Direct, Proquest, and Embase databases. See Supplementary Material for the search strategy. Third, Google and Google scholar were searched for gray literature. Fourth, search results of all databases collected in the EndNote X7 software and duplicate articles were removed.

### Study Criteria

Studies were individually screened in two steps: screening titles and abstracts and screening full-text articles by two investigators using eligibility criteria. Articles with these characteristics were selected to review (a) randomized controlled trials; (b) study participants, including the elderly without Alzheimer, cognitive problems, and impaired mental function; (c) using one or more interventions to improve the elderly's ADL independence; (d) directly or indirectly measuring older people's ability and independence in performing individual or social ADL, and (e) English language studies. The exclusion criteria were non-original articles, including letters to the editor, case reports, review and meta-analysis studies, and articles presented at conferences. It should be noted that articles without full text were excluded.

### Risk of Bias in Included Studies

**Figure 1:**
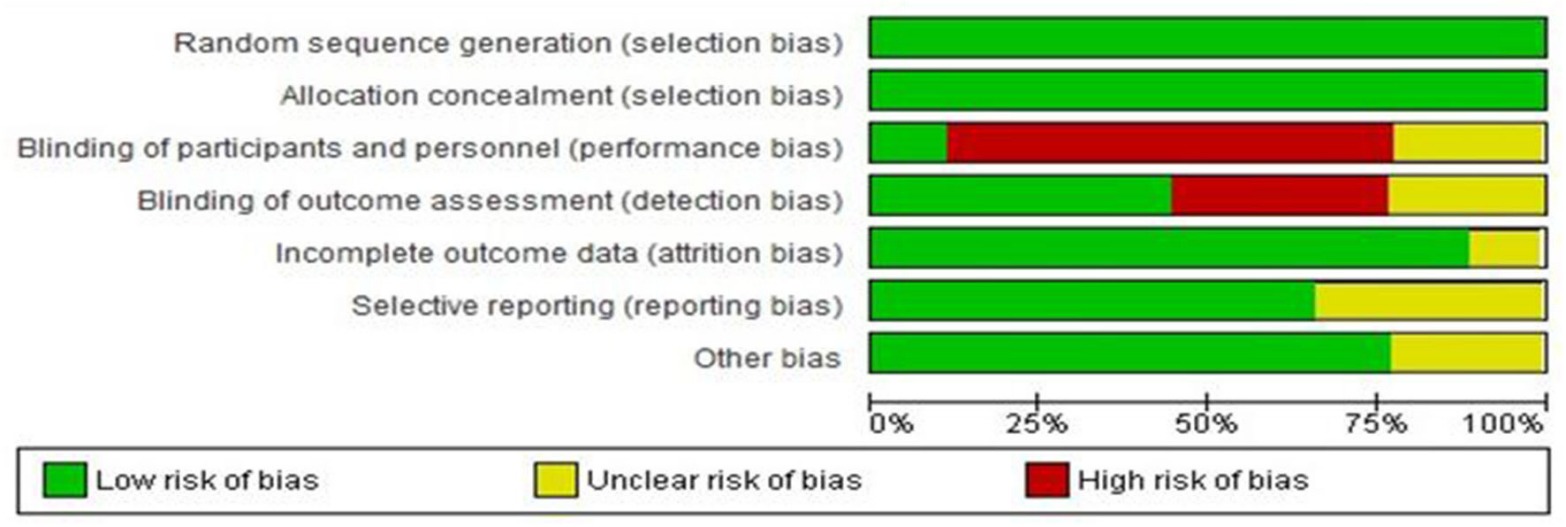
Risk of bias graph: review authors' judgments about each risk of bias item presented as percentages across all included studies.

**Figure 2:**
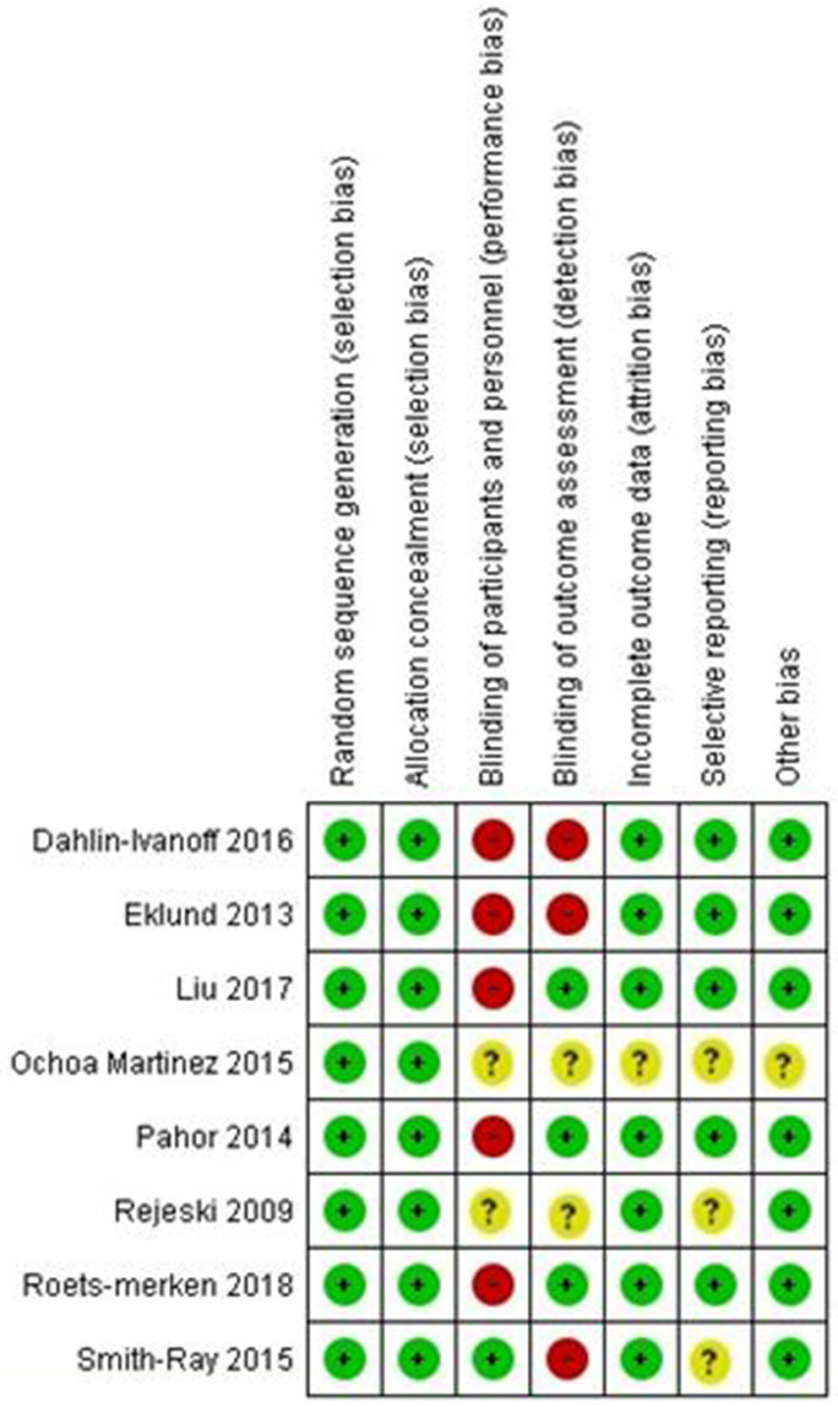
Risk of bias summary: review author's judgments about each risk of bias item for each included study.

Review Manager 5.1 (RevMan) software was used to determine the risk of bias. Details of the risk-of-bias items are presented separately in each article in Figures 1, 2.

#### Data Synthesis

We did not perform a meta-analysis due to heterogeneity in quantitative indices and outcome measurement tools. Instead, we chose a narrative approach to synthesis according to the steps outlined in the Center for Reviews and Dissemination Guidance: developing a theory of how the intervention works, why and for whom, developing a preliminary synthesis of results of included studies, exploring relationships in the data, considering the robustness of the synthesis (21).

## Results

### Description of Studies

**Figure 3:**
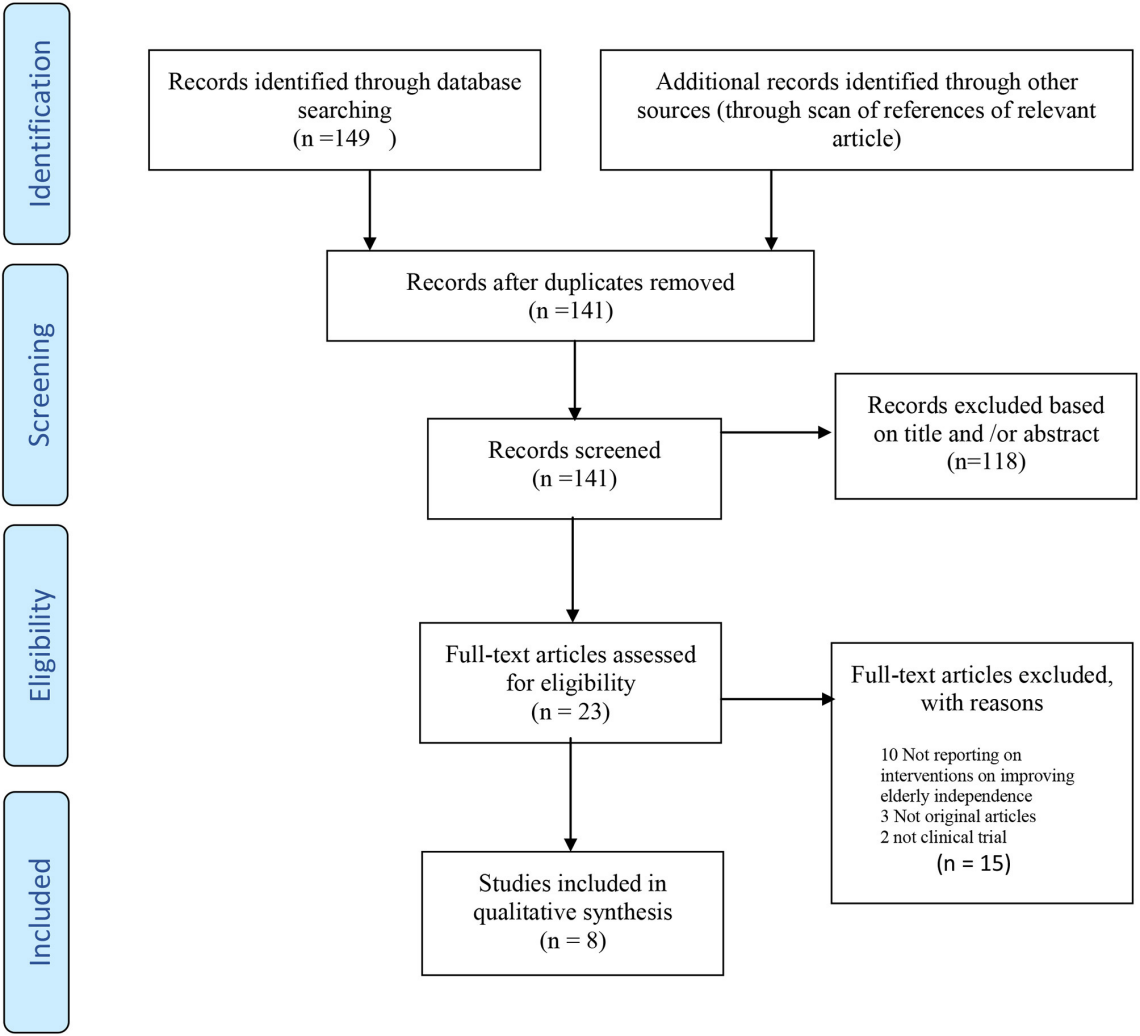
Search process and study identification systematic review of studies with a theoretical framework during interventions for improving elderly's independence in ADLs.

A total of 343 articles were found after searching the database. Then, 212 articles remained after removing the duplicate articles. After reviewing the articles' titles and abstracts, 33 articles entered the next stage. At this stage, the articles' full texts were reviewed, and eight RCT studies entered the final analysis. Several articles were excluded in the screening stages of studies for various reasons, such as non-relevant topics, non-interventional subjects, and non-original articles. The flowchart of the studies entered is shown in Figure 3.

### Study Characteristic

Eight eligible randomized controlled trials were conducted in four different countries, including the USA (four studies), Sweden (two studies), Netherland (one study), and Mexico (one study) (22–29). The sample size varied from 26 (25) to 1,635 participants (26), with an average of 354 participants. The interventions' duration varied from 10 weeks (24, 29) to 12 months (26, 27). The follow-up time range varied from a very brief follow-up (29) to 36 months (27). The characteristics of the studies are summarized in Table 1.

**Table 1:** Summary of the included RCT studies.

**Results**	**ADL or independence scales**	**Duration month**	**Interventions to maintain independence**	**Country/Year**	**Authors**
SMP was more effective in IADL	**- The Patient Autonomy Questionnaire (PAQ) (****30****)** - **Activity Card Sort (ACS) (****31****):**-4 domains: Instrumental activities of daily living (IADL) Social-cultural activities High-physical-demand leisure activities Low-physical-demand leisure activities.	5	- **Self-management program (SMP) (****32****):** - Problem-solving approach−5 steps: Problem identification Collecting alternatives Choice and planning Execution Reflection	Netherlands 2018	Roets-Merken et al. (28)
3-Step Workout for Life improves the performance of ADLs for older adults who are at risk of losing independence at home.	- **Assessment of Motor & Process Skills (AMPS):** - A standardized observational evaluation - Evaluation of 16 motor skills and 20 process skills of familiar ADL	2.5	- **The 3-Step Workout for Life program (****24****):** - Task-oriented approach−3 components: Muscle strength training Functional training ADL training	USA 2017	Liu et al. (24)
There is a positive effect of ADL independence in the intervention group	- **ADL staircase (****33****):** - Evaluation of personal and instrumental ADL(10 original activities) - A scoring range of independence to dependency	—-	- **Elderly Persons in the Risk Zone (EPRZ):** - Empowerment approach−2 interventions due to maintaining independence: Preventive home visit (PHV) Senior group meetings (SM).	Sweden 2016	Dahlin-Ivanoff et al. (22)
Maintaining balance and gait through improving working memory and speed processing.	- **Measurement of ADL through a demographic questionnaire** - **Berg Balance Scale** - Functional balance test	2.5	- **A computer-based cognitive training program “Insight”:**-3 simple computer games target executive function domains - A self-driven program that adapts to the individual's performance by increasing or decreasing task difficulty	USA 2015	Smith-Ray et al. (29)
Positive improvements in functional autonomy	- **5 tests to determine the functional autonomy according to GDLAM protocol** 10 m walk (10 mW) Getting up from a seated position (GSP) Getting up from the prone position (GPP) Getting up from a chair and movement around the house (GCMH) Putting on and taking off a shirt (PTS)	3	- **Water exercise training program:** - Five times a week, with 50 min per session for 3 months - Providing functional autonomy (34)	Mexico 2015	Ochoa Martínez et al. (25)
Reducing major mobility disability in activity daily living	- **Short Physical Performance Battery (SPPB)** - Measurement of walking, balance, and strength tasks - Score ranging from worst performance to best performance - prediction of mobility disability and ADL disability	12	- **The Lifestyle Interventions and Independence for Elders (LIFE):** - The physical activity (PA) intervention along with group-mediated behavioral counseling sessions focusing on self-regulatory skills (35)	USA 2014	Pahor et al. (26)
Promoting ADL independence up to 1 year and postponing ADL dependence up to 6 months	- **ADL staircase** - Evaluation of ADL - A scoring range of independence to dependency	6	- **Continuum of Care for Frail Older People:** - An integrated care and rehabilitation by a multi-professional team from hospital to homes	Sweden 2013	Eklund et al. (23)
Better SPPB and walking speed	- **Short Physical Performance Battery (SPPB)**	12	- **The Lifestyle Interventions and Independence for Elders (LIFE)** - Similar to the study of Pohar et al. 2014 (26)	USA 2009	Rejeski et al. (27)

Based on the overall key risk of bias items, seven (87%) of eight articles were rated as low risk of bias (studies with three or more items were considered low risk). Seven (87%) studies were identified as high or unclear risk of performance, indicating that participants were not blind in five studies, and in two studies, their blindness was unclear. In three studies, the assessor was not blind, there was an unclear risk of detection bias in two articles, and attrition rates were clearly reported in seven articles (Figure 2).

Different interventional programs have been used in selected articles to promote the elderly's independence that can be divided into three categories of cognitive, physical, and multicomponent interventions. Two of the eight articles utilized cognitive interventions to improve ADLs, including “the self-management program” (SMP) (28) and “cognitive training programs and the computer software program, Insight” (29). Two articles focused on the elderly's physical training intervention, which included the three-step workout for life (24) and the water exercise training program (25). Four articles also used three multicomponent interventions, including the Elderly Persons in the Risk Zone (EPRZ) (22), “Lifestyle and Independence Interventions for the Elderly” (LIFE) (26, 27), and a Continuum of Care for Frail Older People (23).

Between cognitive interventions, SMP focused on improving the elderly's IADLs with five skills: problem-solving, decision-making, resource utilization, formulation of participation, and actions (28). The insight program was another cognitive intervention that emphasized promoting BADLs by improving executive function, including working memory, processing speed, and inhibition, using three different games: Road Tour, Jewel Diver, and Sweep Seeker (29).

As a physical training intervention, a three-step workout for life program focused on slowing down disability in the elderly and improving performance in ADLs. In this exercise, functional movements, selective daily activities, and resistance exercises were used three times a week, 60 min each time, for 10 weeks (24). The other physical intervention was water exercise training, which revealed significant improvement in the elderly's functional independence. Five water exercises were performed in this intervention, including walking 10 meters, standing up from a chair and walking straightaway, dressing and undressing, standing up from a sitting position, and standing up from a lying position (25).

One of the multicomponent interventions was the LIFE protocol consisting of cognitive and physical interventions (26, 27). The LIFE protocol had several components: social cognitive theory (self-efficacy and outcome expectations) (36), aerobics, endurance exercises, and flexible training activities. This program was finally able to improve BADLs (18). The EPRZ program focused on the elderly's independence in ADL and encouraged the elderly to decide independently and gain control over their lives and learn how to turn their home into an age-friendly environment (22). “Continuum of care for frail older people” was a program that concentrated on interprofessional shared decision-making for continuous care (23). The independence of the elderly in this program was improved in five BADLs (bathing, dressing, going to the toilet, transferring, and feeding) and four IADLs (cleaning, buying, transporting, and cooking) (33).

## Discussion

This systematic review provides available evidence about the impact of cognitive, physical, and multicomponent interventions on the elderly's independence in IADLs and BADLs.

Cognitive programs sought to prevent the reduction of executive functions and other aspects of working memory that is damaged during the aging process (37). According to the results, cognitive interventions had two outcomes: improving independence in IADLs and BADLs. As a cognitive program, SMP strengthened the elderly's independence in IADLs by improving internal locus of control, participation, problem-solving, and self-determination skills (28). It is justified in light of the self-efficacy theory that the internal control mechanism can gradually control the individual's behaviors and allows the person to adapt to the social and physical environment (38). Rebok et al. also showed that active cognitive training effectively improved IADLs in the elderly for 10 years (12). The “Insight program” enhanced BADLs in the elderly by using computer games stimulated parts of the brain that controls movements (29). This program therefore focused on executive functions that include selective attention and working memory (39). Selective attention can solve the problems of essential cognitive ability and improve BADLs (40). Researchers in a study stated that video game training could significantly enhance the elderly's cognitive and physical performance, independence, and right to choose (41). The weakness of these two studies was the lack of evaluation of IADLs and BADLs simultaneously.

The results showed that the outcome of physical intervention was the improvement of independence in BADLs. The “three-step workout for life program” (24) and “water exercise training program” (25) focused on physical activity and improving familiar and simple activities for promoting the elderly's independence in BADLs. Physical exercise is an efficient and cost-effective way to prevent the loss of the elderly's functional ability (42). Physical activity's positive effects include independence in self-care activities, higher self-esteem, better life quality, higher life expectancy, and lower mortality (43). Some studies state that exercise can delay elderly dependence and improve physical performances, such as sitting and standing, maintaining balance, and movement (44, 45). Chou et al. support this finding that exercise training interventions are beneficial to boost walking speed and improve balance and performance in the elderly's ADLs (10).

Multicomponent interventions had a variety of consequences. LIFE intervention improved BADLs (26, 27) and focused on psychological empowerment, concepts of Bandura's social cognitive theory (46), and a group-mediated approach (47). This method leads to the internalization of the locus of control (48). The results of Blankevoort et al.'s study support this finding and indicate multicomponent interventions can improve the elderly's physical performance and BADLs (49). Bandura's cognitive theory justifies how enhancing self-efficacy beliefs and outcome expectations in the elderly increases their self-confidence and sense of control over life and make them more independent (50). According to these results, in two programs of the “continuum of care for frail older people” (23) and the “EPRZ” (51), researchers have been able to purposefully improve the independence of the elderly in both IADLs and BADLs. It should be noted that tailoring interventions can be designed as cognitive, physical, or combined interventions based on independence in IADLs or BADLs. Also, the emphasis on creating an age-friendly environment is very valuable in EPRZ because it is made in accordance with the restrictions of the elderly and preserves their independence. In a study consistent with this finding, researchers stated that combined interventions should be tailored based on the elderly's needs and preferences to build a safe and independent life for them (18).

### A Proposed Logic Model for Designing Intervention

**Figure 4:**
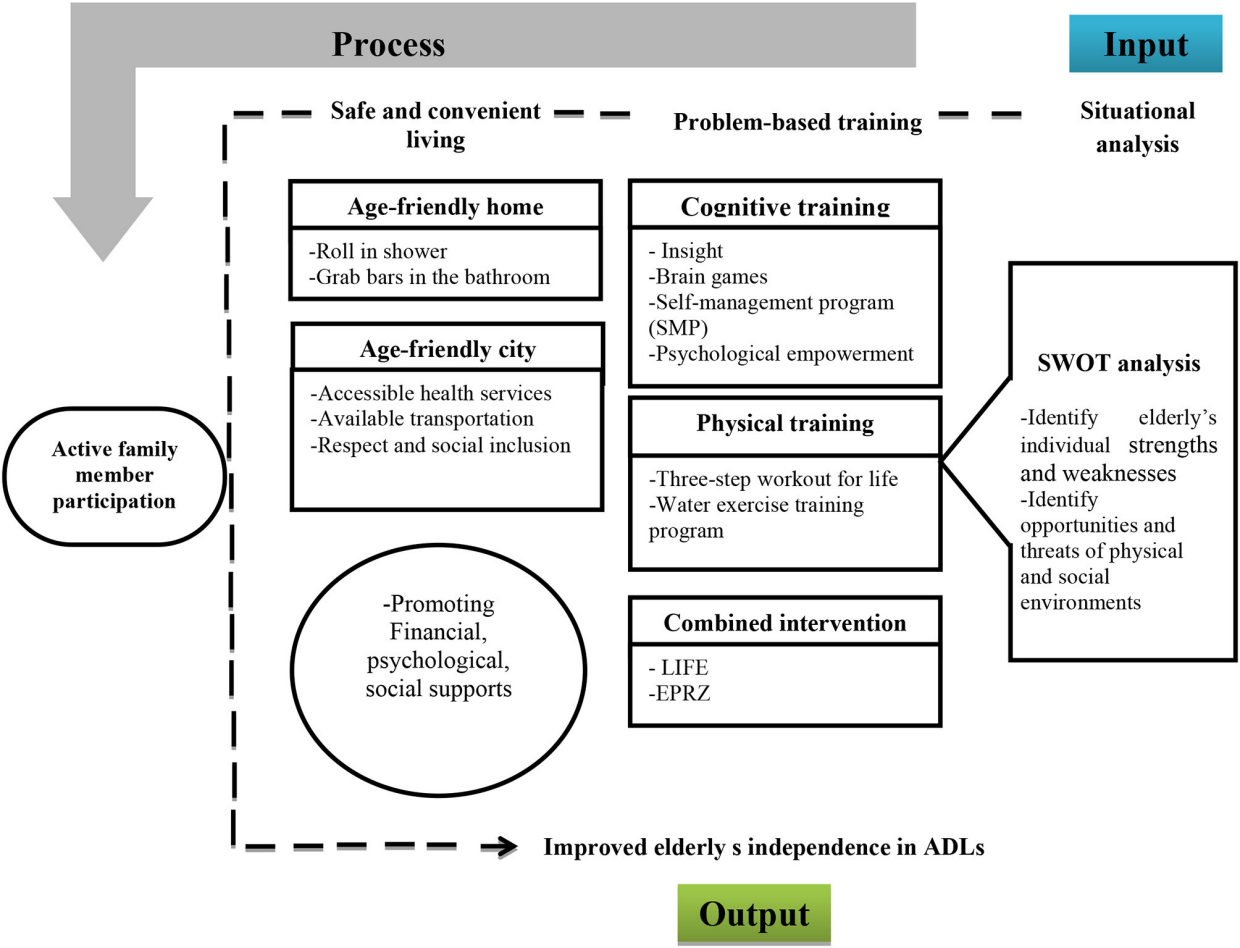
Developing a logic model for improving elderly's independence in ADLs.

Given that independence has different dimensions and is known as a multifactorial phenomenon, it requires tailored multifaceted interventions (52, 53). It seems that the design and evaluation of multicomponent interventions using standard protocols in different elderly populations should be considered in future studies. On the other hand, individual socioeconomic differences among the elderly indicate that tailoring interventions are necessary. Therefore, inspired by the logical models in health promotion program planning, we developed a conceptual framework for designing multicomponent interventions to promote the elderly's independence in activities of daily living. The main components of our proposed logic model are plotted in Figure 4 and described below.

In the input, situational analysis performs. In our logic model, situation analysis refers to analyzing a collection of the elderly's individual strengths and weaknesses and opportunities and threats of physical and social environments (SWOT analysis).

The process section shows the methods and activities designed to implement the interventions and provide services to the target audience (54). According to the situational analysis, this phase uses problem-based interventions, including cognitive, physical, and multicomponent interventions. Creating an age-friendly environment is also considered to maintain the elderly's independence in our proposed model. According to the WHO, health is affected by how people live, and the physical and social environment can affect people's health-oriented behavior (55). Therefore, it is necessary to design cities and elderly's homes under age-friendly environments. The third component of the process is the need for economic, psychological, and social support to sustain the elderly's independence, which can be achieved through family, friends, NGOs, and the government.

The output demonstrates the achievements of a program in the target audience (56), and it was specified in our logic model as having improved the elderly's independence in ADLs.

## Conclusion

This systematic review provides a useful overview of effective interventions to improve the elderly's independence in ADLs. The development of a conceptual framework is the novelty of this study, which provides a better insight to design interventions for the elderly's independence. We recommend the use of standard protocols for the design, implementation, and evaluation of interventions because it can help to better compare interventions in systematic review and meta-analysis studies. Our proposed logic model can, therefore, be tested as a guiding framework in the design of interventions. Another systematic review should also be performed—one that focuses on a specific type of intervention, such as cognitive interventions.

### Limitation

In its binding to English-language publications, the studies' geographical scope has been limited. Also, the studies done in Persian but not indexed in English databases were not included in the strategy search. In the present study, the meta-analysis was not performed due to shortcomings in quantitative indices and different measuring tools.

## Data Availability Statement

All datasets generated for this study are included in the article/Supplementary Material.

## Author Contributions

MK and MM-J collaborated equally on all parts of this manuscript.

## Conflict of Interest

The authors declare that the research was conducted in the absence of any commercial or financial relationships that could be construed as a potential conflict of interest.
