# Supplementary material for: Effective Interventions on Improving Elderly's Independence in Activity of Daily Living: A Systematic Review and Logic Model
Source: Front Public Health. 2021 Feb 15;8:516151. doi: 10.3389/fpubh.2020.516151 (PMC7917261; doi:10.3389/fpubh.2020.516151)
Supplement: Supplementary file 1 [file Table_1.docx]

| Table 1. Summary of the included RCT studies | | | | | |
| --- | --- | --- | --- | --- | --- |
| **Results** | **ADL or independence scales** | **Duration**  **Month** | **Interventions to maintain independence** | **Country/ Year** | **Authors** |
| SMP was more effective in IADL | **-The Patient Autonomy Questionnaire (PAQ)(57)**  -**Activity Card Sort (ACS)(58):**  -4 domains:   1. Instrumental activities of daily living (IADL) 2. Social-cultural activities 3. High-physical-demand leisure activities 4. Low-physical-demand leisure activities. | 5 | **-Self-management program (SMP)(59):**  -Problem-solving approach  -5 steps:   1. Problem identification 2. Collecting alternatives 3. Choice and planning 4. Execution 5. Reflection | Netherlands  2018 | Roets-Merken et al. |
| 3-Step Workout for Life improves the performance of ADLs for older adults who are at risk of losing independence at home. | **-Assessment of Motor & Process Skills (AMPS):**  -A standardized observational evaluation  -Evaluation of 16 motor skills and 20 process skills of familiar ADL | 2.5 | -**The 3-Step Workout for Life program(24):**  -Task-oriented approach  -3 components:   1. Muscle strength training 2. Functional training 3. ADL training | USA  2017 | Liu et al. |
| There is a positive effect of ADL independence in the intervention group | **- ADL staircase(36):**  -Evaluation of personal and instrumental ADL(10 original activities)  -A scoring range of independence to dependency | ---- | -**Elderly Persons in the Risk Zone (EPRZ):**  -Empowerment approach  -2 interventions due to maintaining independence:   1. Preventive home visit (PHV) 2. Senior group meetings (SM). | Sweden  2016 | Dahlin-Ivanoff et al. |
| Maintaining balance and gait through improving working memory and speed processing. | **-Measurement of ADL through a demographic questionnaire**  **-Berg Balance Scale**  -Functional balance test | 2.5 | **-A computer-based cognitive training program "Insight":**  -3 simple computer games target executive function domains  -A self-driven program that adapts to the individual's performance by increasing or decreasing task difficulty | USA  2015 | Smith-Ray et al. |
| Positive improvements in functional autonomy | **-5 tests to determine the functional autonomy according to GDLAM protocol**   1. 10 m walk (10 mW) 2. Getting up from a seated position (GSP) 3. Getting up from the prone position (GPP) 4. Getting up from a chair and movement around the house (GCMH) 5. Putting on and taking off a shirt (PTS) | 3 | **-Water exercise training program :**  - Five times a week, with 50 minutes per session for 3 months  - Providing functional autonomy(60) | Mexico  2015 | Ochoa Martínez et al. |
| Reducing major mobility disability in activity daily living | **-Short Physical Performance Battery (SPPB(**  -Measurement of walking, balance, and strength tasks  -Score ranging from worst performance to best performance  -prediction of mobility disability and ADL disability | 12 | **-The Lifestyle Interventions and Independence for Elders (LIFE) :**  -The physical activity (PA) intervention along with group-mediated behavioral counseling sessions focusing on self-regulatory skills(61) | USA  2014 | Pahor et al. |
| Promoting ADL independence up to 1 year and postponing ADL dependence up to 6 months | **-ADL staircase**  -Evaluation of ADL  -A scoring range of independence to dependency | 6 | **-Continuum of Care for Frail Older People:**  -An integrated care and rehabilitation by a multi-professional team from hospital to homes | Sweden  2013 | Eklund et al. |
| Better SPPB and walking speed | **-Short Physical Performance Battery (SPPB)** | 12 | **-The Lifestyle Interventions and Independence for Elders (LIFE)**  -Similar to the study of Pahor et al. (2014) | USA  2009 | Rejeski et al. |

-Accessible health services

-Available transportation

-Respect and social inclusion

**Safe and convenient living**

**Active family member participation**

**Age-friendly city**

**Physical training**

-Three-step workout for life

-Water exercise training program

-Roll in shower

-Grab bars in the bathroom

**Age-friendly home environment**

**Combined intervention**

- Insight

-Brain games

-Self-management program (SMP)

-Psychological empowerment

**Cognitive training**

**Input**

**Process**

**Situational analysis**

**Problem-based training**

**SWOT analysis**

-Identify elderly’s individual strengths and weaknesses

-Identify opportunities and threats of physical and social environments

- LIFE

-EPRZ

**Improved elderly s independence in ADLs**

**Output**

**Figure 4:** Developing a logic model for improving elderly’s independence in ADLs
