# Supplementary material for: Effective Interventions on Improving Elderly's Independence in Activity of Daily Living: A Systematic Review and Logic Model
Source: Front Public Health. 2021 Feb 15;8:516151. doi: 10.3389/fpubh.2020.516151 (PMC7917261; doi:10.3389/fpubh.2020.516151)
Supplement: Supplementary file 2 [file Table_2.docx]

**Search strategy without time limit up to 16th October 2019**

| pubmed | | |
| --- | --- | --- |
| 1 | (((aged[MeSH Terms]) OR frail elderly[MeSH Terms]) OR (age,80 and over[MeSH Terms]) Filters: Full text elder* or Older adults) | [299315](https://www.ncbi.nlm.nih.gov/pubmed/?cmd=HistorySearch&querykey=1) |
| 2 | |  | ((independence[Title/Abstract]) OR self-determination[Title/Abstract]) OR autonomy[Title/Abstract] | | --- | --- | | [68559](https://www.ncbi.nlm.nih.gov/pubmed/?cmd=HistorySearch&querykey=2) |
| 3 | ((cognitive interventions[Title/Abstract]) OR physical interventions[Title/Abstract]) OR environmental interventions[Title/Abstract] | [1584](https://www.ncbi.nlm.nih.gov/pubmed/?cmd=HistorySearch&querykey=3) |
| #1 AND #2 AND #3 | |  | ((((((aged[MeSH Terms]) OR frail elderly[MeSH Terms]) OR (age,80 and over[MeSH Terms]) Filters: Full text elder* or Older adults))) AND (((independence[Title/Abstract]) OR self-determination[Title/Abstract]) OR autonomy[Title/Abstract])) AND (((cognitive interventions[Title/Abstract]) OR physical interventions[Title/Abstract]) OR environmental interventions[Title/Abstract]) Filters: Clinical Trial; Humans | | --- | --- | | 2 |

PROQUEST

Additional limits - Source type: Scholarly Journals; Document type: Article; Language: English

| **Prequest** | |  |
| --- | --- | --- |
| 1#Abstract | ab(aged) OR ab(elder) OR ab(aged OR elderly) OR ab(old age) | 243,815 |
| 2#Abstract | ab(independence) OR ab(autonomy) OR ab(self-determination) | 16,659 |
| 3#Abstract | ab(cognitive interventions) OR ab(physical interventions) OR ab(environmental interventions) | 32,267 |
| 1 AND #2 AND #3 ANDAny where | (ab(cognitive interventions) OR ab(physical interventions) OR ab(environmental interventions)) AND (ab(aged) OR ab(elder) OR ab(aged OR elderly) OR ab(old age)) AND (ab(independence) OR ab(autonomy) OR ab(self-determination)) | 277 |

| **SCOPUS** | |  |
| --- | --- | --- |
| 1 | |  | ( TITLE-ABS-KEY ( elderly )  OR  TITLE-ABS-KEY ( aged )  OR  TITLE-ABS-KEY ( age,80 )  OR  TITLE-ABS-KEY ( older )  OR  TITLE-ABS-KEY ( geriatric ) ) | | --- | --- | | | [6,583,875](https://www.scopus.com/search/history/results.uri?origin=searchhistory&shid=1) |  | | --- | --- | |
| 2 | ( TITLE-ABS-KEY ( independence )  OR  TITLE-ABS-KEY ( self-determination )  OR  TITLE-ABS-KEY ( autonomy ) ) | [263,300](https://www.scopus.com/search/history/results.uri?origin=searchhistory&shid=2) |
| 3 | ( TITLE-ABS-KEY ( cognitive  AND interventions )  OR  TITLE-ABS-KEY ( physical  AND interventions )  OR  TITLE-ABS-KEY ( environmental  AND interventions ) ) | [219,190](https://www.scopus.com/search/history/results.uri?origin=searchhistory&shid=3) |
| 4 | ( TITLE-ABS-KEY ( randomised  AND clinical  AND trial )  OR  TITLE-ABS-KEY ( rct ) ) | [710,179](https://www.scopus.com/search/history/results.uri?origin=searchhistory&shid=7) |
| 5 | ( TITLE-ABS-KEY ( activity  AND daily  AND living )  AND  TITLE-ABS-KEY ( adl ) ) | [10,568](https://www.scopus.com/search/history/results.uri?origin=searchhistory&shid=10) |
| Combine#1 AND #2 AND #3 AND #4 AND #5 | ( ( TITLE-ABS-KEY ( elderly ) OR TITLE-ABS-KEY ( aged ) OR TITLE-ABS-KEY ( age,80 ) OR TITLE-ABS-KEY ( older ) OR TITLE-ABS-KEY ( geriatric ) ) ) AND ( ( TITLE-ABS-KEY ( independence ) OR TITLE-ABS-KEY ( self-determination ) OR TITLE-ABS-KEY ( autonomy ) ) ) AND ( ( TITLE-ABS-KEY ( cognitive AND interventions ) OR TITLE-ABS-KEY ( physical AND interventions ) OR TITLE-ABS-KEY ( environmental AND interventions ) ) ) AND ( ( TITLE-ABS-KEY ( randomised AND clinical AND trial ) OR TITLE-ABS-KEY ( rct ) ) ) AND ( ( TITLE-ABS-KEY ( activity AND daily AND living ) AND TITLE-ABS-KEY ( adl ) ) ) AND ( LIMIT-TO ( DOCTYPE , "ar" ) ) | 36 |

| Science Direct | |  |
| --- | --- | --- |
| filter: Research articles | Title, abstract, keywords: (Self-determination or autonomy) and (elderly or aging or geriatric) | 1 |

|  | **Embase** |  |
| --- | --- | --- |
|  | ((aged:ab,ti OR 'frail elderly':ab,ti OR age,80:ab,ti) AND over:ab,ti OR 'older adults':ab,ti) AND ('cognitive interventions':ab,ti OR 'physical interventions':ab,ti OR 'environmental interventions':ab,ti) AND (independence:ab,ti OR autonomy:ab,ti OR 'self determination':ab,ti) AND [randomized controlled trial]/lim AND [1966-2019]/py | 4 |

| Cochrane Trials | |  |
| --- | --- | --- |
|  | **22 Trials matching Self-determination in Title Abstract Keyword AND elderly in Title Abstract Keyword - (Word variations have been searched)** | 22 |

**Total =342**
